# A LRRK2 GTP Binding Inhibitor, 68, Reduces LPS-Induced Signaling Events and TNF-α Release in Human Lymphoblasts

**DOI:** 10.3390/cells10020480

**Published:** 2021-02-23

**Authors:** Tianxia Li, Bo Ning, Lingbo Kong, Bingling Dai, Xiaofei He, Joseph M. Thomas, Akira Sawa, Christopher A. Ross, Wanli W. Smith

**Affiliations:** 1 Department of Psychiatry and Behavioral Sciences, Johns Hopkins University School of Medicine, Baltimore, MD 21287, USA; Tianxia_Li@DFCI.HARVARD.EDU (T.L.); bning2@jhmi.edu (B.N.); lingbokong@hotmail.co.uk (L.K.); dbl1412@xjtu.edu.cn (B.D.); hexiaof2@mail2.sysu.edu.cn (X.H.); asawa1@jhmi.edu (A.S.); caross@jhu.edu (C.A.R.); 2 Department of Pharmaceutical Sciences, University of Maryland School of Pharmacy, Baltimore, MD 21201, USA; j.m.thomas35@gmail.com; 3 Departments of Neurology, Neuroscience and Pharmacology, Johns Hopkins University School of Medicine, Baltimore, MD 21287, USA; 4 Departments of Psychiatry, Neuroscience, Biomedical Engineering, and Genetic Medicine, Johns Hopkins University School of Medicine, Baltimore, MD 21205, USA; 5 Department of Mental Health, Johns Hopkins University Bloomberg School of Public Health, Baltimore, MD 21205, USA

**Keywords:** LRRK2, GTP binding inhibitor, LPS, TNF-α, lymphoblast, Parkinson’s disease

## Abstract

Mutations in *the leucine-rich repeat kinase-2 (LRRK2)* gene cause autosomal-dominant Parkinson’s disease (PD) and contribute to sporadic PD. Common genetic variation in LRRK2 modifies susceptibility to immunological disorders including Crohn’s disease and leprosy. Previous studies have reported that LRRK2 is expressed in B lymphocytes and macrophages, suggesting a role for LRRK2 in immunological functions. In this study, we characterized the LRRK2 protein expression and phosphorylation using human lymphoblasts. Lipopolysaccharide (LPS), a proinflammatory agent, induced the increase of LRRK2 expression and kinase activities in human lymphoblasts in a time-dependent manner. Moreover, LPS activated the Toll-like receptor (TLR) signaling pathway, increased TRAF6/LRRK2 interaction, and elevated the phosphorylation levels of MAPK (JNK1/2, p38, and ERK1/2) and IkBα. Treatment with LRRK2 inhibitor 68 reduced LPS-induced TRAF6/LRRK2 interaction and MAPK and IkBα phosphorylation, thereby reducing TNF-α secretion. These results indicate that LRRK2 is actively involved in proinflammatory responses in human lymphoblasts, and inhibition of GTP binding by 68 results in an anti-inflammation effect against proinflammatory stimuli. These findings not only provide novel insights into the mechanisms of LRRK2-linked immune and inflammatory responses in B-cell-like lymphoblasts, but also suggest that 68 may also have potential therapeutic value for LRRK2-linked immunological disorders.

## 1. Introduction

Parkinson’s disease (PD) is a common neurodegenerative disease, with clinical features including tremor, rigidity, bradykinesia, and postural instability [1,2]. The key pathological features of PD include loss of dopamine neurons in the substantia nigra, and the presence of Lewy bodies and chronic neuroinflammation in the brain. Mutations in the *leucine-rich repeat kinase 2 (LRRK2)* gene cause familial PD with clinical and pathological features resembling sporadic PD, likely via genetic gain-of-function (via activating point mutations) [3,4,5,6]. Furthermore, genetic variation at the LRRK2 locus is an important contributor to otherwise apparently “sporadic” PD, also likely via gain-of-function mechanisms. The most prevalent mutation, G2019S-LRRK2, causes about 1% of idiopathic PD in North America and 5% of familial PD [3,4,5,6]. LRRK2 is expressed in neurons and in immune cells, including B lymphocytes, macrophages, and microglia [4,7,8]. LRRK2 can be identified in Lewy bodies [9]. The normal functions of LRRK2 are still unclear [4,7].

Recent studies suggest that LRRK2 plays important roles in innate immunity and neuroinflammation underlying PD-linked neurodegeneration [10,11,12]. Neuroinflammation has been suggested to play a role in the progression of neurodegenerative diseases, including LRRK2-linked PD [10,13,14]. Both PD patients and animal models of PD display increased inflammatory responses, including microglia activation [10,13,14], and an increase of inflammatory cytokines in the brain, cerebrospinal fluid, and blood of PD patients [10,13,14,15]. The *LRRK2* locus is implicated in immune-related disorders, such as Crohn’s disease and leprosy [16,17,18,19,20]. Downregulation of LRRK2 expression or inhibition of LRRK2 kinase and GTP binding activities modulates neuroinflammation and inflammatory cytokine production [21,22,23,24], suggesting that LRRK2 may play an important role in inflammatory-related innate immune responses [15,25]. However, the underlying mechanism of the LRRK2-mediated inflammatory response has not yet been clearly elucidated.

Inflammation has been suggested to play a role in the progression of neurodegenerative diseases, including PD [15,25]. However, the mechanisms of inflammation in PD pathogenesis, especially LRRK2-linked PD, are still poorly understood. B lymphocyte (B cell) is one of the major immune response cells. It functions in the immunity component of the adaptive immune system. LRRK2 is highly expressed in B lymphocytes [26,27], suggesting a role for LRRK2 in regulating B cell functions. LRRK2 is a large protein (2527 amino acid residues) and has GTP binding and kinase activities that may contribute to PD pathogenesis [3,4,5,6,28,29]. We previously demonstrated that LRRK2 kinase activity contributes to mutant LRRK2-mediated cellular toxicity [28], and we have defined the role of GTPase domain activity [21,22,30,31]. We and others have found that inhibition of GTP binding and kinase activities suppresses mutant LRRK2-induced neurodegeneration and other related pathologies [22,28,32,33,34,35].

We recently identified a novel LRRK2 GTP binding inhibitor, compound 68, which potently inhibits both LRRK2 GTP binding and kinase activities in cultured cells in vitro, and provides a useful pharmacological tool for studying LRRK2 functions [21,22,30,31]. Here, we use 68 to study whether inhibition of LRRK2 GTP binding activity regulates human lymphoblast responses to proinflammatory stimuli, lipopolysaccharide (LPS).

## 2. Materials and Methods

### 2.1. Reagents

Compound 68 was ordered from Chembridge Corporation (San Diego, CA, USA) [21,22]. Compound 68 was dissolved in 0.1% DMSO for use in cell cultures and biochemical assays. LPS was purchased from Sigma (St. Louis, MO, USA). Anti-phospho-S935 antibody and anti-LRRK2 antibodies were from the Michael J. Fox Foundation (MJFF). Antibodies against total and phosphorylated JNK1/2, ERK1/2, p38, 4EBP, and IKBa were purchased from Cell Signaling Technology (Beverly, MA, USA). Anti-actin antibody (clone Y-11) was purchased from Santa Cruz (Dallas, TX, USA). Alexa Fluor 568 goat anti-rabbit antibody and RPMI 1640 media were from Invitrogen (Carlsbad, CA, USA). The FITC goat anti-mouse antibody was from Millipore (Billerica, MA, USA).

### 2.2. Human Lymphoblast Culture and Treatment

Human lymphoblasts were generated from normal human blood lymphocytes at the core facility, Johns Hopkins University, as described previously [36] by Dr. Akira Sawa’s group. Human lymphoblasts were grown in RPMI 1640 media containing 15% fetal bovine serum (FBS) and an antibiotic mixture. This study was conducted in accordance with the Declaration of Helsinki, and the protocol was approved by the Ethics Committee of Johns Hopkins University School of Medicine (Project identification code, NA-00037204). All human subjects gave their informed consent for inclusion before they participated in the study as described in Dr. Sawa’s previous paper [36]. In this study, we only used lymphoblasts (without the identification information of human subjects) to perform in vitro studies. LPS (1 µg/mL) was added to cell media for 10 min to 3 days. Compound 68 (10 nM) was pre-treated 2 h before LPS exposure and then kept treatment for 10 min to 3 days.

### 2.3. Western Blot and Co-Immunoprecipitation (Co-IP) Assays

Cells were harvested in lysis buffer (Cell Signaling Technology, Beverly, MA, USA) with protease inhibitors (10 µg/mL aprotinin/5 mM PMSF/10 µg/mL pepstatin/10 µg/mL leupeptin) as described previously [29]. The resulting lysates were subjected to co-IP and Western blot analysis. Co-IP experiments were performed using anti-LRRK2 antibodies (5 µL per reaction) and protein G–agarose (GE, Pittsburgh, PA, USA) as described previously [29]. For Western blot analysis, proteins were separated on 4–12% NuPAGE Bis-Tris gels for 1–3 h, and transferred onto polyvinylidene difluoride (PVDF) membranes. The PVDF membranes were incubated with 4% non-fat milk for 1 h, followed by the addition of various primary antibodies as listed in the materials section. The goat anti-rabbit or goat anti-mouse horseradish-peroxidase-conjugated antibodies were used as secondary detection antibodies. Then, the PVDF membranes were incubated with enhanced chemiluminescence reagents (PerkinElmer, Waltham, MA, USA) for 1 min to detect the proteins in each lane. The quantification of phosphorylated protein levels was normalized to total protein and/or internal control actin levels. The ratio of phosphorylated to total protein and/or actin was calculated. The fold increase of phosphorylation was calculated relative to untreated control cells.

### 2.4. Immunocytochemical Analysis

Human lymphoblasts were placed in slides to dry overnight and subjected to immunocytochemical analysis as described previously [29]. Cells were probed with anti-CD19 (a B-cell marker) and anti-LRRK2 antibodies, followed by incubation with the secondary antibodies: Alexa Fluor 568 goat anti-rabbit antibody and FITC goat anti-mouse antibody. Nuclei were stained with DAPI (4′,6-diamidino-2-phenylindole). The images were captured by confocal fluorescent microscopy (LSM 510 and Axiovert 100, Zeiss).

### 2.5. TNF-α Measurement

Culture supernatants from lymphoblasts were collected to measure TNF-α using a commercial ELISA kit (Invitrogen, Carlsbad, CA, USA) according to the manufacturer’s instructions using anti-TNF-α antibodies. Three replicates in each sample were included in each experiment. Three independent experiments were performed. TNF-α levels were normalized to protein levels.

### 2.6. Data Analysis

The experiments were repeated at least three times. Quantitative data are presented as means ± standard error of mean (SEM). Comparisons of protein and TNF-α levels were used for one-way or two-way analysis of variance (ANOVA), followed by Tukey’s post-hoc test using Sigmastart 3.1 statistical software (Aspire Software International, VA, USA). A *p* value < 0.05 was considered significant.

## 3. Results

### 3.1. Inhibition of GTP Binding by 68 Reduced LPS-Induced LRRK2 Kinase Activation

**Figure 1:**
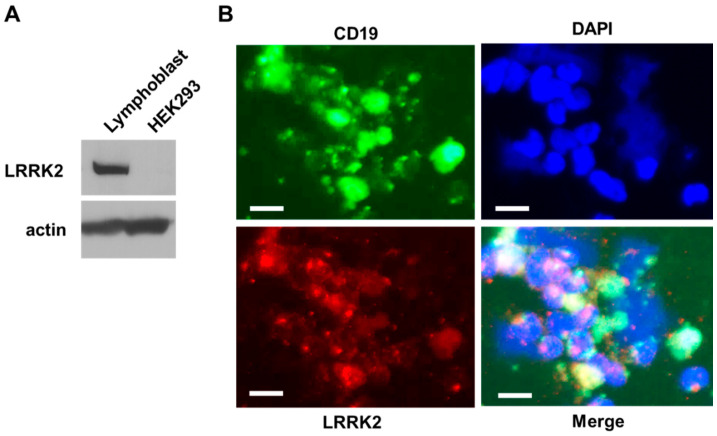
Leucine-rich repeat kinase-2 (LRRK2) is expressed in human lymphoblasts. (**A**) Cell lysates from human lymphoblasts and HEK293T cells were subjected to immunoblot analysis using anti-LRRK2 (top) antibodies. Equal protein input was controlled by a Western blot using anti-actin (bottom). (**B**) Representative co-immunostained images of human lymphoblasts are shown using anti-CD19 (B-cell marker, green) and anti-LRRK2 (red) antibodies. DAPI was used to label nuclei. The white scale bar indicates 10 microns.

To study the roles of LRRK2 in response to LPS, we used the human lymphoblasts that were generated by Dr. Akira Sawa’s group from blood lymphocytes of normal human subjects as described previously [36]. We found that endogenous LRRK2 was highly expressed in lymphoblasts (Figure 1a). Moreover, there were over 95% of these lymphoblasts immunostained with anti-CD19 antibodies (a B-cell marker) and co-localized with anti-LRRK2 immunostaining in some degree (Figure 1b). These results indicate that these lymphocytes predominantly were B cells, which provided a useful cell system to study the roles of LRRK2 in response to LPS proinflammatory stimuli.

**Figure 2:**
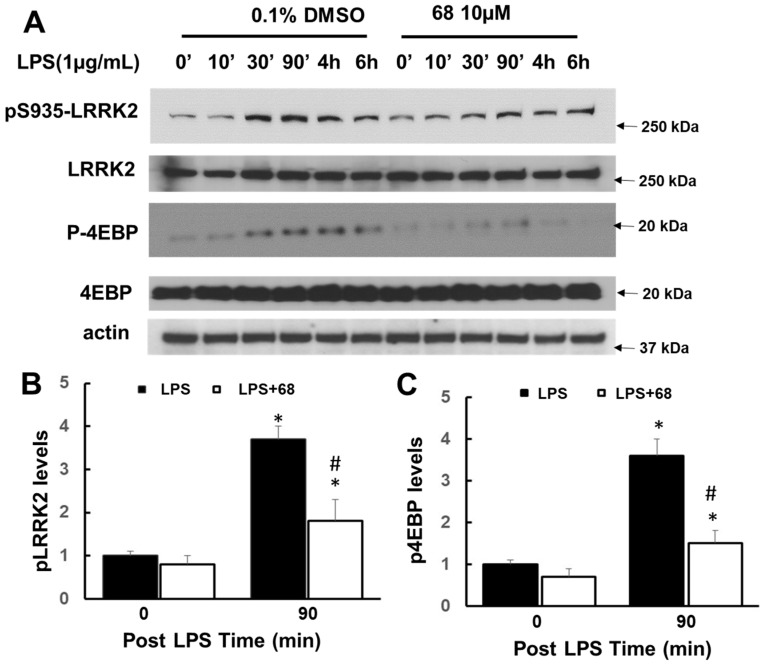
Compound 68 reduced lipopolysaccharide (LPS)-induced LRRK2 kinase activity. Human lymphoblasts were incubated in serum-free medium for 12 h and then exposed to LPS (1 µg/mL) with or without pre-treating 68 (10 µM) for 2 h, then kept treatment for various time periods as indicated. Cell lysates were harvested at the indicated time points after LPS exposure, then were subjected to immunoblot analysis using the indicated antibodies. (**A**) Representative blots from at least three separate experiments. (**B**,**C**) The quantification of phosphorylated pLRRK2 (**B**) and p4EBP (**C**) levels at 90 min, peak time. * *p* < 0.05 by ANOVA, vs. untreated cells. # *p* < 0.05 by ANOVA, vs. cells at 90 min post LPS exposure.

Exposure to LPS significantly increased LRRK2 phosphorylation with the peak time at 90 min and slightly increased LRRK2 expression (Figure 2a). LPS induced an increase of LRRK2 phosphorylation over 3 folds compared with those of control cells (Figure 2a,b). Moreover, LPS also increased 4EBP phosphorylation (Figure 2a,c), which is a downstream effector of LRRK2 kinase. Pretreatment with a LRRK2 GTP binding inhibitor, 68, attenuated LPS-induced LRRK2 and 4EBP phosphorylation compared with vehicle control cells (Figure 2).

### 3.2. Compound 68 Reduced LPS-Induced Tumor Necrosis Factor-α (TNF-α) Secretion

**Figure 3:**
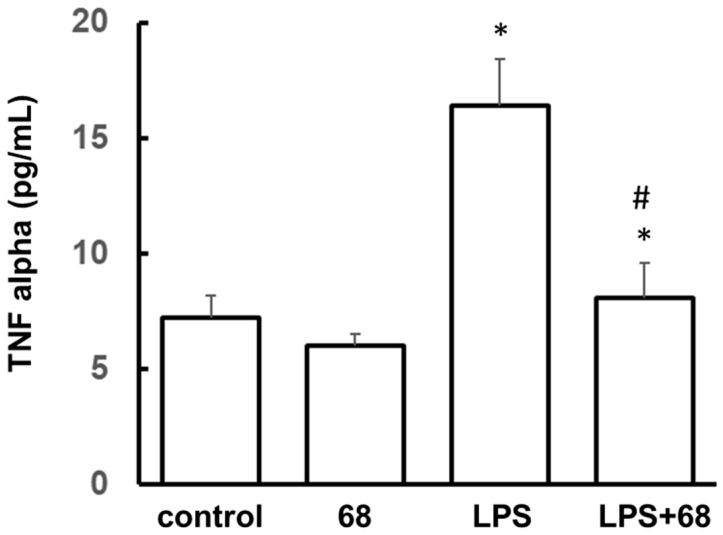
Compound 68 reduced LPS-induced TNF-α secretion. Human lymphoblasts were exposed to LPS (1 µg/mL) with or without 68 (10 µM) pretreatment for 2 h, then kept treatment for 72 h. The supernatants of culture media from each group were collected and subjected to ELISA to detect TNF-α. The TNF-α levels in each group were normalized to the total protein content from each group. * *p* < 0.05 by ANOVA, vs. untreated cells. # *p* < 0.05 by ANOVA, vs. cells with LPS exposure.

LPS can induce immune cells to release TNF-α (a cytokine), thereby contributing to inflammatory response [37,38]. Consistent with these previous studies, exposure to LPS significantly increased TNF-α release in the supernatants of lymphoblasts compared with control cells (Figure 3). Pretreatment with 68 slightly reduced TNF-α release in no-LPS exposure control lymphoblasts compared with the vehicle treatment control group. Importantly, 68 strikingly attenuated LPS-induced TNF-α release. These results suggest that inhibition of LRRK2 GTP binding plays a critical role in inflammatory response and cytokine release.

### 3.3. Compound 68 Reduced LPS-Induced TRAF6/LRRK2 Interaction

**Figure 4:**
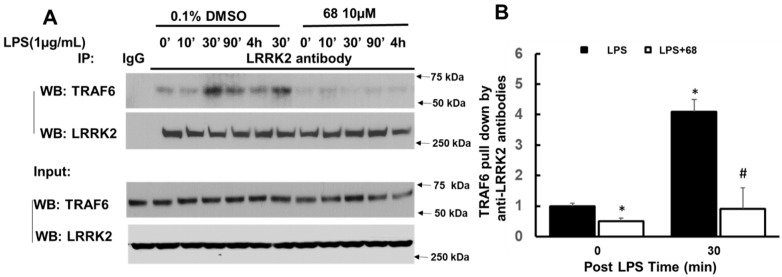
Compound 68 reduced LPS-induced TRAF6 and LRRK2 interaction. Human lymphoblasts were incubated in serum-free medium for 12 h and then exposed to LPS (1 µg/mL) with or without pre-treating 68 (10 µM) for 2 h, then kept treatment for various time periods as indicated. Cell lysates were harvested at the indicated time points after LPS exposure, then were subjected to co-IP assays using an anti-LRRK2 antibody, followed by anti-TRAF6 and anti-LRRK2 immunoblotting. LRRK2 antibody pulled down TRAF6. LPS increased LRRK2 interaction with TRAF6, while 68 attenuated this interaction. (**A**) Representative blots from the three separate experiments. (**B**) The quantification of LRRK2 interaction with TRAF6 at 30 min post LPS exposure (peak time). * *p* < 0.05 by ANOVA, vs. untreated cells at 0 min # *p* < 0.05 by ANOVA, vs. cells at 30 min post LPS exposure.

Previous studies demonstrated that LPS can activate the Toll-like receptor (TLR)-linked signaling pathway in B cells [37,38]. LPS stimulates and activates TLR to form a protein complex containing TNF receptor-associated factor 6 (TRAF6), which then can trigger mitogen-activated protein kinase (MAPK) and/or IkBα-linked NF-kB pathways to regulate cytokine production [39,40]. Our results show that LPS increased TRAF6 interaction with LRRK2 by co-IP assays using lysates of lymphoblasts (Figure 4). This is consistent with the previous studies showing LRRK2 interaction with TRAF6 in the immune reaction [41,42]. Interestingly, 68 significantly attenuated LPS-induced LRRK2 interaction with TRAF6 compared with those of the vehicle-treated control group (Figure 4).

### 3.4. Compound 68 Attenuated LPS-Induced Activation of TRAF6-Linked MAPK and IkBα Signaling Pathways

**Figure 5:**
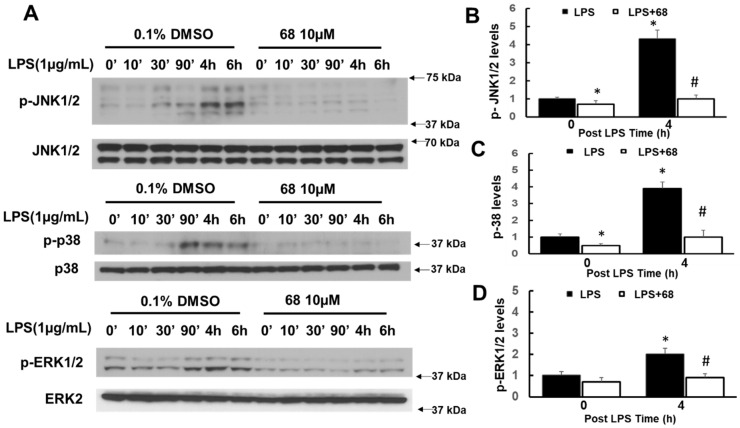
Compound 68 reduced LPS-induced TRAF6-linked MAPK signaling pathways. Human lymphoblasts were incubated in serum-free medium for 12 h and then exposed to LPS (1 µg/mL) with or without pre-treating 68 (10 µM) for 2 h, then kept treatment for various time periods as indicated. Cell lysates were harvested at the indicated time points after LPS exposure, then were subjected to immunoblot analysis using indicated antibodies. (**A**) Representative blots from the three separate experiments. (**B**–**D**). The quantification of phosphorylated pJNK 1/2 (**B**), p38 (**C**), and pERK1/2 levels at peak time. * *p* < 0.05 by ANOVA, vs. untreated cells at 0 min. # *p* < 0.05 by ANOVA, vs. cells at 4 h post LPS exposure.

To further study the LRRK2 roles and 68 action, we investigated whether 68 alters the two downstream pathways of TRAF6-complex, MAPK, and IkBα. We found that LPS activated MAPK signaling pathways, which include three distinct sub-signaling pathways, c-Jun-N-terminal kinase 1/2 (JNK1/2), p38, and extracellular signal-regulated kinase 1/2 (ERK1/2). LPS increased the phosphorylation of JNK1/2, p38, and ERK1/2 (Figure 5). Treatment with 68 attenuated LPS-induced phosphorylation of JNK1/2, p38, and ERK1/2 compared with LPS exposure control cells (Figure 5).

**Figure 6:**
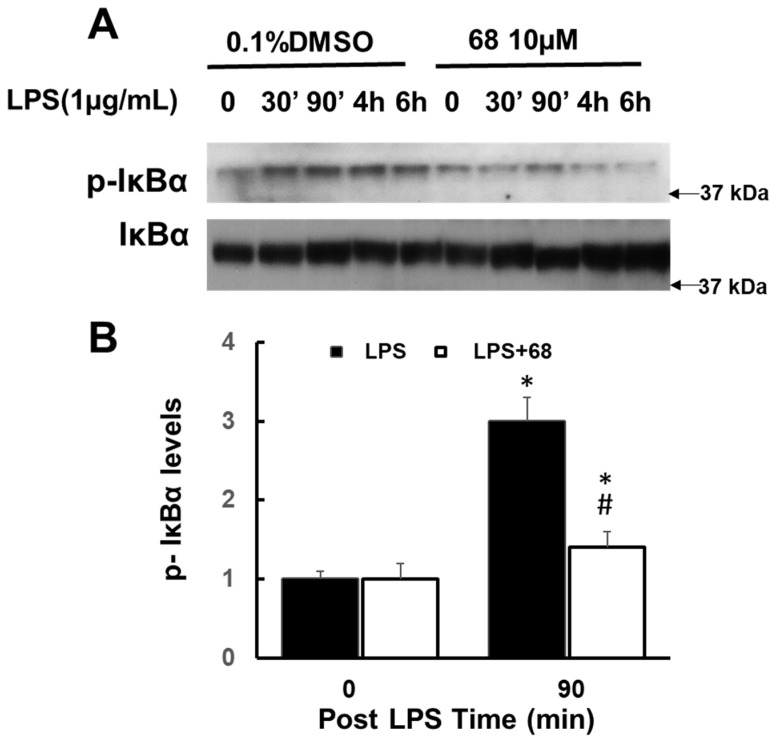
Compound 68 reduced LPS-induced TRAF6-linked IKBa phosphorylation. Human lymphoblasts were incubated in serum-free medium for 12 h and then exposed to LPS (1 µg/mL) with or without pre-treating 68 (10 µM) for 2 h, then kept treatment for various time periods as indicated. Cell lysates were harvested at the indicated time points after LPS exposure, then were subjected to immunoblot analysis using indicated antibodies. (**A**) Representative blots from the three separate experiments. (**B**) The quantification of phosphorylated pIKB levels at 90 min post LPS exposure (peak time). * *p* < 0.05 by ANOVA, vs. untreated cells at 0 min, # *p* < 0.05 by ANOVA, vs. cells at 90 min post LPS exposure.

Moreover, we also found that LPS increased IkBα phosphorylation while 68 reduced this increase (Figure 6) compared with vehicle control cells. The peak times of MAPK and IkBα phosphorylation (90 min to 6 h post LPS exposure) were behind the peak time of TRAF6 interaction with LRRK2 (30 to 90 min post LPS exposure). These findings further demonstrate that TRAF6 and LRRK2 interaction is a critical early event for LPS-linked signaling pathways.

## 4. Discussion

**Figure 7:**
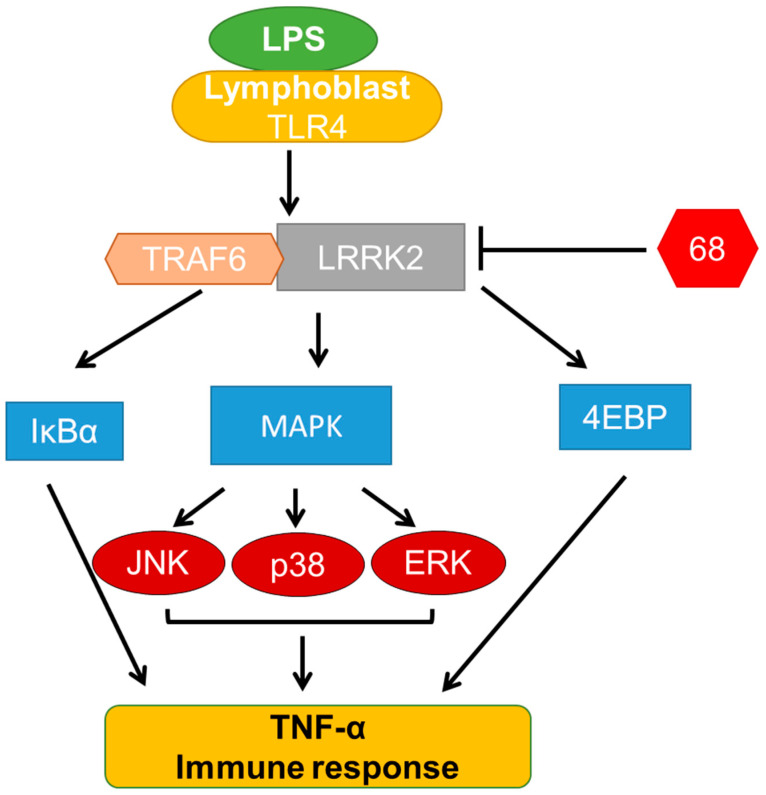
Proposed 68 action model in LPS-induced signaling pathways and TNF-α release.

In this study, we demonstrated that pharmacological inhibition of GTP binding by 68 attenuated LPS-induced signaling events and TNF-α release. LPS activated TLR receptors, resulting in the increase of TRAF6 and LRRK2 interaction. Moreover, the increase of this interaction triggered the activation of MAPK (JNK1/2, p38, and aERK1/2) and IkBα signaling events, thereby leading to an increase of TNF-α release. Inhibition of LRRK2 GTP binding attenuated the interaction between TRAF6 and LRRK2, and blocked the downstream MAPK and IkBα signaling to reduce the TNF-α release. The actions of 68 in LPS-linked signaling pathways and in TNF-α release are shown as the proposed model in Figure 7. These findings demonstrate that LRRK2 GTP binding activity is critical in response to inflammatory stimuli and regulates the immune functions of B-cell-like human lymphoblasts.

The inflammatory response and inflammation are important contributing factors to the PD pathogenesis [15,25]. Immune cytokines (e.g., TNF-α, interleukins) are elevated in the brain, blood, and cerebrospinal fluid of PD patients [10,13,14,15]. During the progression of PD, neurotoxic molecules are released within neurons, which can trigger inflammatory signaling cascades, resulting in excessive inflammation that causes further neural degeneration. LRRK2 is involved in the inflammatory response induced by lipopolysaccharide (LPS) [43,44]. Rats deficient in LRRK2 are protected against LPS-induced dopaminergic neurodegeneration [45]. Moreover, LPS enhances LRRK2 expression and activity [21,22]. There is an increase of TNF-α expression and the cell death in the microglial cells from LRRK2-R1441G transgenic mice after LPS stimulation [46]. Consistently, our results found that LPS exposure increased LRRK2 phosphorylation and TNF-α release in B-type lymphoblasts, while treatment with 68 significantly blocked LPS-induced LRRK2 activation and TNF-α release. These results indicate that inhibition of LRRK2 GTP binding acts as an anti-inflammation effect in B-cell-like lymphoblasts.

Recent studies suggest that the signaling mechanisms link between LRRK2 and innate immunity and inflammatory response are associated with the TLR-linked signaling pathway. Activation of the TLR by ligands, LPS, or other inflammatory stimuli results in the formation of the receptor complex containing TRAF6 [47]. Then, the TRAF6-linked complex dissociated from the receptor, and activated the downstream signaling pathways, including MAPK and NF-kB pathways, thereby increasing the cytokine release and regulating the immune response. Previous studies showed that LRRK2 interacted with a TRAF6-linked complex [41,42]. Our results demonstrate that LPS increased the interaction of LRRK2 and TRAF6. In contrast, 68 significantly reduced this increase of interaction. Moreover, 68 also attenuated the LPS-induced MAPK (JNK1/2, p38, and ERK1/2) and IkBα phosphorylation, which are the downstream effectors in the TLR signaling pathways. These findings indicate that inhibition of LRRK2 GTP binding by 68 could block LPS-induced TLR signaling pathways and, therefore, control of the immune response. In line with this notion, other studies suggest LRRK2-linked MAPK and IkBα pathways in neurodegeneration and neuroinflammation [48,49]. For instance, we and others previously demonstrated that PD-linked LRRK2 mutants can activate the JNK pathway (one sub-signaling pathway of MAPK) in vitro and in vivo, and that blockade of the JNK pathway can protect against mutant LRRK2-induced neurodegeneration [48,49]. In addition, LRRK2 is involved in regulated ERK5 and IκB kinase (IKK)-linked pathways [50]. Interferon-γ increases LRRK2 activation through ERK5 signaling [50]. Treatment with IKK inhibitors inhibits LPS-induced LRRK2 phosphorylation in vitro [51]. LRRK2 deletion enhances the phosphorylation of NF-κB-inhibitory subunit p50 at S337 and NF-κB accumulation in the nucleus [52]. Moreover, LRRK2 appears to positively regulate NF-kB-dependent gene transcription [53]. Taken together, these findings suggest that LRRK2 can regulate MAPK and IkBα signaling in various types of cells (neuron, microglia, and B cells) and results in the regulation of different cellular functions (neurodegeneration, neuroinflammation, peripheral immune response, etc.).

Compound 68 is a potent and relatively specific GTP binding inhibitor for LRRK2, which did not alter LRRK1 and other small GTPase activities [22]. Previously, we found that 68 can reduce LRRK2 kinase activity and protects against mutant LRRK2-induced neuron degeneration in vitro [22]. Here, our results show that 68 significantly blocked LPS-induced B-cell signaling pathways and reduced TNF-α release in lymphoblasts. This is the first report showing that inhibition of LRRK2 GTP binding by 68 can inhibit the LPS-induced proinflammatory response. Our study may provide the proof-of-principle that 68 can also be used as an anti-inflammation agent, and a potential lead compound for further anti-inflammatory drug development. Moreover, targeting LRRK2 GTP binding activity to combat PD not only reduces neurodegeneration, but also attenuates inflammation in brains and in immune cells, which are key pathological features in PD.

In conclusion, our findings demonstrate that LRRK2 GTP binding inhibitor 68 attenuated LPS-induced signaling events and TNF-α release. These studies not only provide novel insight into the mechanisms of LRRK2-linked immune and inflammatory responses in B-type lymphoblasts, but also provide a potential anti-inflammatory action of compound 68. Besides LRKK2 inhibition and neuron protective effects, 68 may also have potential therapeutic value for LRRK2-linked PD and immunological disorders.

## Data Availability

All data are included in the paper.
